# Clinical features and KRAS mutation in colorectal cancer with bone metastasis

**DOI:** 10.1038/s41598-020-78253-x

**Published:** 2020-12-03

**Authors:** Hyung Soon Park, You Jin Chun, Han Sang Kim, Jee Hung Kim, Choong-kun Lee, Seung-Hoon Beom, Sang Joon Shin, Joong Bae Ahn

**Affiliations:** 1 Department of Internal Medicine, College of Medicine, The Catholic University of Korea, Seoul, Korea; 2 Division of Medical Oncology, Department of Internal Medicine, St. Vincent’s Hospital, The Catholic University of Korea, Suwon, Korea; 3 Division of Medical Oncology, Department of Internal Medicine, Yonsei University College of Medicine, Seoul, Korea; 4 Division of Medical Oncology, Department of Internal Medicine, Gangnam Severance Hospital, Yonsei University College of Medicine, Seoul, Korea

**Keywords:** Cancer, Oncology

## Abstract

Bone metastasis is known as a poor prognostic factor in colorectal cancer (CRC), but its clinical manifestations and outcomes are uncertain. CRC with bone metastasis was searched from January 2006 to April 2016. Of 11,551 CRC patients, 321 (2.7%) patients had bone metastasis. Bone-only metastasis was found in only 8.7% of patients. Synchronous bone metastasis was present in 147 (45.8%) patients. In patients with metachronous bone metastasis, the median time from CRC diagnosis to bone metastasis (TTB) was 27.2 months. KRAS mutation status was a marginally significant factor affecting TTB (median TTB, KRAS wild-type or mutation: 29 or 25.8 months, respectively, P = 0.068). Skeletal-related events (SREs) were noted in 200 (62.3%) patients. Median overall survival (OS) from diagnosis of bone metastasis was 8.0 months. On multivariate analysis, multi-organ metastasis, peritoneal metastasis, neutrophil-to-lymphocyte ratio (NLR) ≥ 2.7, and alkaline phosphatase (ALP) ≥ 123 were independent factors for OS. Palliative chemotherapy prolonged survival in CRC patients with bone metastasis (HR 0.25, 95% CI 0.2–0.33). In conclusion, bone metastasis of CRC is rare, but it is related to SREs. Most patients have other organ metastasis and survival is 8.0 months. Attention should be paid to bone metastasis in CRC patients.

## Introduction

Colorectal cancer (CRC) was the fourth most common cancer in the United States, with a total of 101,420 cases of newly diagnosed CRC in 2019^1^. The most common metastatic sites were liver (70%) and lung (37%)^2^. Bone metastasis was observed in 1.2–12% of patients with CRC. The ten-year incidence of bone metastasis from CRC has been reported to be 2.7%^3,4^. Recently, bone metastasis incidence has increased due to improvement of survival of patients with metastatic CRC and diagnostic imaging techniques^5^. Bone metastasis of CRC is related to poor survival outcome. The median survival of patients with bone metastasis of CRC ranges from 7 months to 9.4 months^6–8^. Previous studies have reported that multiple bone metastases, lung metastasis, and elevated carcinoembryonic antigen (CEA) are poor prognostic factors in CRC patients. However, these factors have not been universally observed in previous reports. The prognostic factors of patients with bone metastasis are still uncertain. A prognostic model has not been suggested yet. In addition, the role of palliative chemotherapy in CRC patients with bone metastasis has been rarely studied.

KRAS mutations have been detected in 40–50% of patients with CRC^9,10^. KRAS mutations at codon 12 or 13 account for approximately 90% of all mutation types. In general, clinical behaviors are more aggressively presented in patients with KRAS mutations. Recurrence-free survival is significantly worse and lung metastasis is significantly higher in CRC patients with KRAS mutations^11–13^. Although the prognostic value of KRAS mutations is still controversial, some studies have revealed that KRAS mutations are associated with poorer survival^12,14–16^. However, associations between KRAS mutations and bone metastases have not been reported yet.

Thus, the aim of this study was to explore the clinical features of CRC patients with bone metastasis, including time to bone metastasis (TTB), metastatic sites of bone, and skeletal-related events (SREs) by KRAS mutation status. In addition, prognostic factors for CRC with bone metastasis were evaluated, and a prognostic model for survival was also suggested.

## Results

### Patient characteristics

Of 11,551 CRC patients, colon cancer (7242, 62.7%) and rectal cancer (4309, 37.3%) with bone metastasis were diagnosed in 192 (2.7%) patients and 129 (3.0%) patients, respectively. Patient characteristics are shown in Table 1. Their median age was 61 years old. There were 188 (58.6%) male patients. Colon cancer was slightly predominant (59.8%). Similar metastatic pattern were observed in both the synchronous and metachronous groups. There were 292 (91%) cases of adenocarcinoma. A total of 229 (71.3%) patients were classified as stage 4 at the initial time of CRC diagnosis. Bone-only metastasis occurred in 28 (8.7%) patients. Frequently observed metastatic sites of bone included the spine (68.8%) and pelvis (51.7%). Median laboratory values of neutrophil-to-lymphocyte ratio (NLR), platelet count, alkaline phosphatase (ALP), and CEA at the time of bone metastasis were 3.5, 247 × 10^3^/μL, 114 IU/L, and 75.4 ng/mL, respectively. Of patients whose KRAS mutation status was revealed, 126 (61.8%) patients had wild-type KRAS, while 78 (38.2%) patients had KRAS mutations. According to the presence of KRAS mutation, old age, females, and right-sided colon cancer patients were statistically predominant in the group with KRAS mutation. In addition, liver metastasis, lung metastasis, high ALP level and high CEA level were frequently observed at the time of bone metastasis in the group with KRAS mutation. Codon 12 mutation, codon 13 mutation, and mutation site unknown accounted for 58, 16, and 4 cases, respectively. Clinical differences by KRAS mutation type and tumor sidedness were also analyzed in Supplementary Tables S1 and S2. A total of 212 (66%) patients received palliative chemotherapy.

**Table 1:** Characteristics of CRC with bone metastasis according to KRAS mutation status.

		All patients		KRAS				
				Wild type		Mutant type		P-value
		No. (321)	%	No. (126)	%	No. (78)	%	
Age	(Median, range)	61	28–87	58	29–83	62	28–79	0.007
Sex	Male	188	58.6	81	64.3	38	48.7	0.028
	Female	133	41.4	45	35.7	40	51.3	
Primary site	Colon	192	59.8	79	62.7	48	61.5	0.868
	Rectum	129	40.2	47	37.3	30	38.5	
Tumor sideness	Left side	250	77.9	105	84.7	55	71.4	0.023
	Right side	63	19.6	19	15.3	22	28.6	
Metastasis pattern	Synchronous	147	45.8	55	43.7	31	39.7	0.583
	Metachronous	174	54.2	71	56.3	47	60.3	
Histologic type	Adenocarcinoma	292	91	114	90.5	73	93.6	0.325
	SRC	14	4.4	7	5.6	1	1.3	
	Mucinous carcinoma	15	4.7	5	4	4	5.1	
Clinical stage at CRC diagnosis	1 or 2	19	5.9	7	5.7	6	7.7	0.283
	3	68	21.2	32	26	13	16.7	
	4	229	71.3	84	68.3	59	75.6	
Bone only metastasis	Yes	28	8.7	9	7.1	2	2.6	0.211
	No	293	91.3	117	92.9	76	97.4	
**Other organ metastasis**								
Liver metastasis	No	137	42.7	59	46.8	22	28.2	0.008
	Yes	184	57.3	67	53.2	56	71.8	
Lung metastasis	No	153	47.7	63	50	28	35.9	0.049
	Yes	168	52.3	63	50	50	64.1	
Peritoneal metastasis	No	243	75.7	90	71.4	60	76.9	0.387
	Yes	78	24.3	36	28.6	18	23.1	
Brain metastasis	No	306	95.3	122	96.8	70	89.7	0.062
	Yes	15	4.7	4	3.2	8	10.3	
**Site of bone metastasis**								
Spine	No	100	31.2	37	29.4	29	37.2	0.246
	Yes	221	68.8	89	70.6	49	62.8	
Pelvis	No	155	48.3	60	47.6	39	50	
	Yes	166	51.7	66	52.4	39	50	
Long bone	No	252	78.5	100	79.4	62	79.5	0.983
	Yes	69	21.5	26	20.6	16	20.5	
Other bone^a^	No	197	61.4	79	62.7	50	64.1	0.84
	Yes	124	38.6	47	37.3	28	35.9	
Laboratory values at diagnosis of bone metastasis (median, range)	NLR	3.5	0.4–31.8	3.5	0.6–19.7	3.6	0.8–31.8	0.937
	Platelet (× 1000/uL)	247	25–654	243	26–637	245	25–654	0.903
	ALP (IU/L)	114	17–1509	98	17–897	136	32–1509	0.025
	CEA (ng/mL)	75.4	0.4–20,000	77.7	0.4–20,000	159.3	2.3–20,000	0.041
Palliative CTx	Irinotecan based	96	29.9	47	37.3	32	41	0.136
	Oxaliplatin based	73	22.7	33	26.2	10	12.8	
	Others^b^	43	13.4	16	12.7	11	14.1	
	No	109	34	30	23.8	25	32.1	

*CRC* colorectal cancer, *CTx* chemotherapy, *NLR* neutrophil–lymphocyte ratio, *SRC* signet ring cell carcinoma, *ALP* alkaline phosphatase, *CEA* carcinoembryonic antigen, *No* number.

^a^Other bone include skull, rib, scapula, knee, clavicle, sternum.

^b^Others include fluoropyrimidine based chemotherapy, target monotherapy (cetuximab, bevacizumab, regorafenib).

### Natural course of bone metastasis

**Figure 1:**
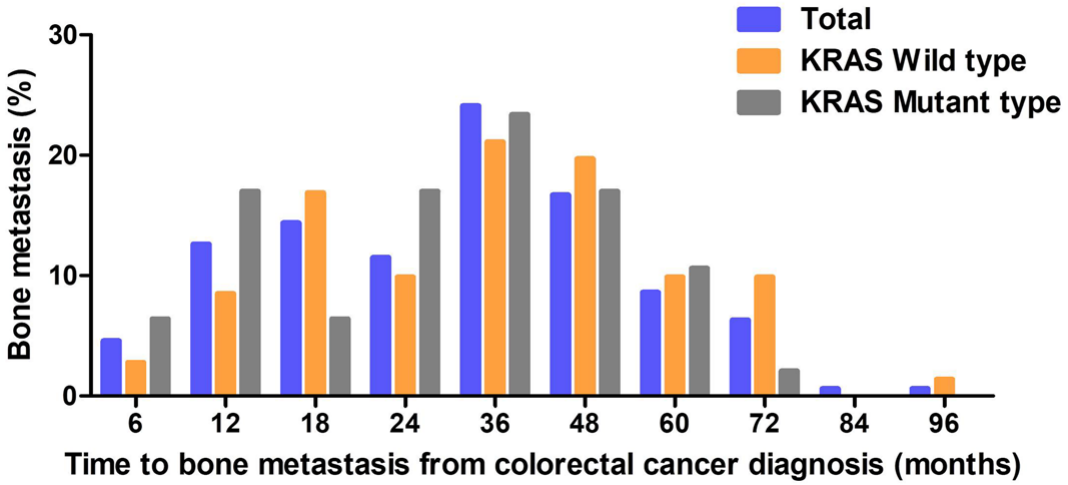
Time to bone metastasis from colorectal cancer (CRC) diagnosis according to KRAS mutation status.

Metachronous bone metastasis occurred in 174 (54.2%) patients (Fig. 1). In metachronous bone metastasis patients, the median TTB was 27.2 months. TTB was analyzed by clinical factors (Table 2). The median TTB in patients with initial clinical stages 1/2, 3, and 4 were 35.9, 28.8, and 20.9 months, respectively (P < 0.001). KRAS mutation status was a marginally significant factor affecting time to bone metastasis. Patients with KRAS wild-type or mutation had a median TTB of 29 months or 25.8 months, respectively (P = 0.068). In addition, mucinous carcinoma had a shorter tendency of time to bone metastasis compared with that of non-mucinous carcinoma (median TTB, 16.2 months vs. 27.4 months, P = 0.269). SREs, pathological fractures, spinal cord compression, surgery to bone, radiotherapy to bone, and hypercalcemia were observed in 26 (8.1%), 28 (87%), 43 (13.4%), 183 (57%), and 12 (3.7%) patients, respectively (Table 3). There was no significant difference in the SRE pattern between patients with KRAS wild-type and patients with KRAS mutation.

**Table 2:** Median time from primary cancer diagnosis to bone metastasis in metachronous patients.

Categorical	No. of patients	Median time (months)	95% CI		P-value
**Age**					
< 61	92	27.4	24.5	30.3	0.171
≥ 61	82	25.7	20.7	30.7	
**Sex**					
Male	106	27.3	23.9	30.7	0.831
Female	68	25.8	20.2	31.4	
**Primary site**					
Colon	91	25.9	21.9	29.9	0.498
Rectum	83	28.3	25	31.6	
**KRAS mutation**					
Wild type	71	29	23.5	34.5	0.068
Mutant type	47	25.8	15.9	35.7	
**Tumor sideness**					
Left side	143	28.3	25.5	31.1	0.457
Right side	29	22.6	14.0	31.2	
**Histologic type**					
Adenocarcinoma or SRC	164	27.4	24.8	30.0	0.269
Mucinous carcinoma	10	16.2	11.1	21.3	
**Initial clinical stage**					
1 or 2	19	35.9	23.2	48.6	< 0.001
3	68	28.8	24.1	33.5	
4	82	20.9	16.3	25.5	
Overall	174	27.2	24.2	30.2	

*SRC* signet ring cell carcinoma, *No* number, *CI* confidence interval.

**Table 3:** Skeletal related events.

Variable	All, No. (321)		KRAS wild type, No. (126)		KRAS mutant type, No. (78)		P-value
	No	%	No	%	No	%	
SRE, total	200	62.3	81	64.3	55	70.5	0.359
Pathologic fracture	26	8.1	12	9.5	6	7.7	0.654
Spinal cord compression	28	8.7	13	10.3	8	10.3	0.989
Surgery to bone	43	13.4	19	15.1	12	15.4	0.953
Radiotherapy to bone	183	57	75	59.5	51	65.4	0.403
Hypercalcemia	12	3.7	5	4	6	7.7	0.34

*SRE* skeletal related event, *No* number.

### Survival analysis

**Figure 2:**
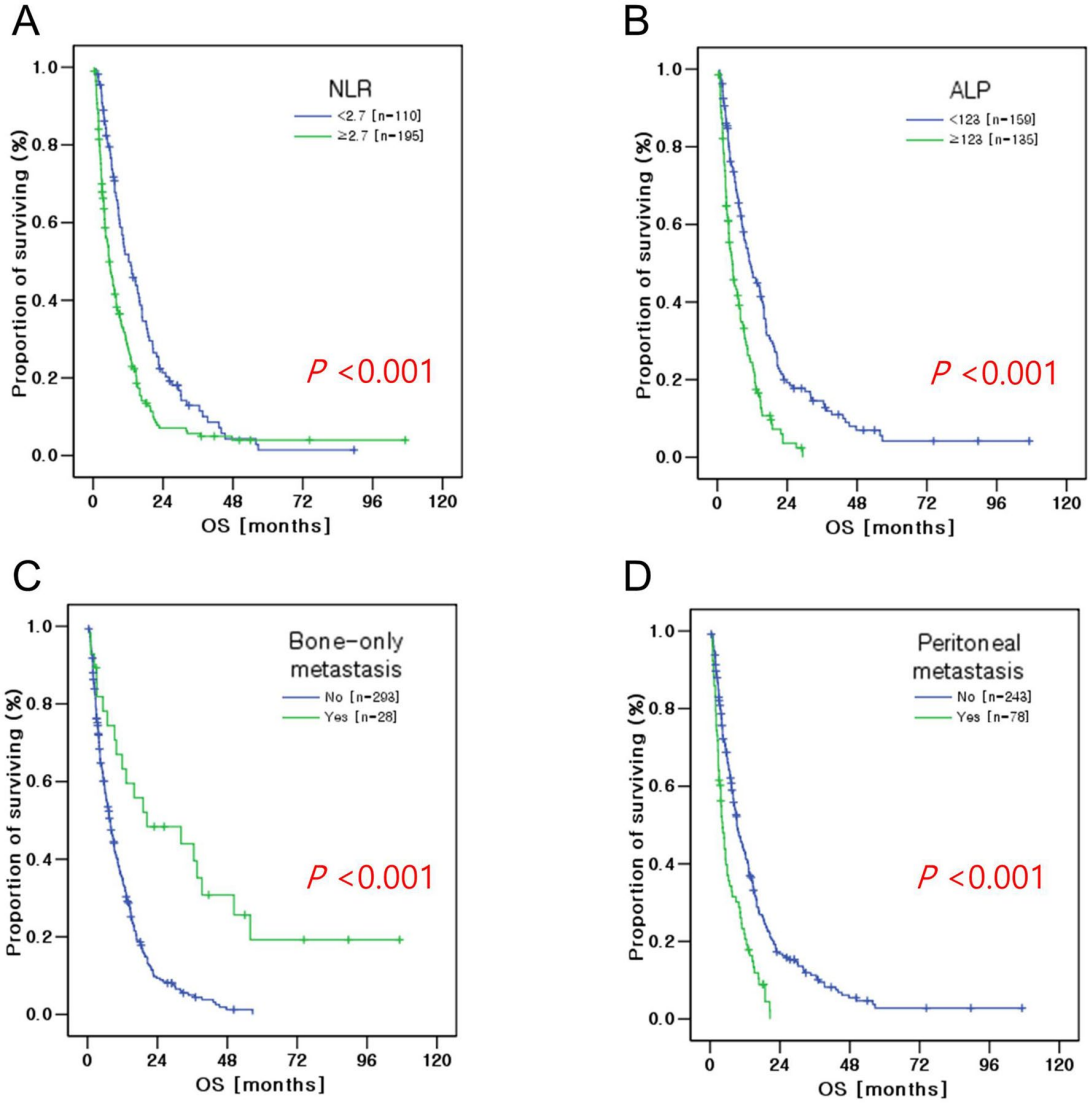
Kaplan–Meier curves of overall survival (OS) for all patients (n = 321) according to prognostic factors: (**A**) neutrophil-to-lymphocyte ratio (NLR), (**B**) alkaline phosphatase (ALP), (**C**) bone-only metastasis, and (**D**) peritoneal metastasis.

The median follow-up time was 7.4 months. The median OS from diagnosis of bone metastasis was 8.0 months (95% confidence interval (CI) 6.8–9.2 months). The statistically determined best cut-off point of NLR was 2.7. Survival was significantly extended in patients with low levels of NLR or ALP compared to patients with high levels of NLR or ALP (median OS, NLR: 12.2 months vs. 5.6 months, P < 0.001; ALP: 11.2 months vs. 5.2 months, P < 0.001; Fig. 2A,B). Patients with bone-only metastasis had the longest median OS of 20.4 months (Fig. 2C). Peritoneal carcinomatosis showed poor survival outcome (median OS: 9.2 months vs. 4.3 months, P < 0.001) (Fig. 2D). However, KRAS mutation status did not significantly affect survival outcome (median OS: wild-type vs. mutant type, 9.2 months vs. 8.4 months, P = 0.083).

According to univariate analysis, primary site; tumor sidedness; bone metastasis pattern (bone-only or multi-organ metastasis); presence of liver, lung or peritoneal metastasis; NLR; ALP level; and CEA level were all associated with OS at p-value < 0.05 (Table 4). Multivariate analysis demonstrated that multi-organ metastasis (Hazard ratio (HR) 2.43; 95% CI 1.39–4.25), peritoneal metastasis (HR 1.47; 95% CI 1.08–2.02), NLR ≥ 2.7 (HR 1.54; 95% CI 1.17–2.04), and ALP ≥ 123 (HR 1.76; 95% CI 1.32–2.34) were independent factors for OS. Bone-only metastasis was a prognostic factor with the highest hazard ratio for OS.

**Table 4:** Univariate and multivariate analysis using cox regression for OS.

Categorical	Univariate					Multivariate			
	No. of patients	HR	95% CI		P-value	HR	95% CI		P-value
**Age**									
< 61	154	1			0.057				
≥ 61	167	1.26	0.99	1.59					
**Sex**									
Male	188	1			0.876				
Female	133	0.98	0.77	1.25					
**Primary site**									
Colon	192	1			0.041				
Rectum	129	0.78	0.61	0.99					
**KRAS mutation**									
Wild type	126	1			0.085				
Mutant type	78	1.31	0.96	1.79					
**Tumor sideness**									
Left side	250	1			0.018				
Right side	63	1.44	1.06	1.94					
**Metastasis pattern**									
Synchronous	147	1			0.815				
Metachronous	174	0.97	0.77	1.23					
**Histologic type**									
Adenocarcinoma	292	1			0.727				
SRC	14	1.15	0.66	2.00					
Mucinous carcinoma	15	0.84	0.48	1.47					
**Bone-only metastasis**									
Yes	28	1			< 0.001	1			0.002
No	293	3.02	1.88	4.87		2.43	1.39	4.25	
**Liver metastasis**									
No	137	1			< 0.001				
Yes	184	1.87	1.45	2.40					
**Lung metastasis**									
No	153	1			0.022				
Yes	168	1.32	1.04	1.67					
**Peritoneal metastasis**									
No	243	1			< 0.001	1			0.016
Yes	78	2.05	1.56	2.71		1.47	1.08	2.02	
**Brain metastasis**									
No	306	1			0.172				
Yes	15	1.46	0.85	2.50					
**NLR**									
< 2.7	110	1			< 0.001	1			0.002
≥ 2.7	195	1.72	1.33	2.21		1.54	1.17	2.04	
**ALP (IU/L)**									
< 123	159	1			< 0.001	1			< 0.001
≥ 123	135	2.17	1.67	2.81		1.76	1.32	2.34	
**CEA (ng/mL)**									
< 5	37	1			< 0.001				
≥ 5	253	2.29	1.52	3.45					
**Palliative CTx**									
No	109	1			< 0.001				
Yes	212	0.25	0.20	0.33					

*NLR* neutrophil–lymphocyte ratio, *ALP* alkaline phosphatase, *CEA* carcinoembryonic antigen, *CTx* chemotherapy, *SRC* signet ring cell carcinoma, *OS* overall survival, *No* number, *HR* hazard ratio, *CI* confidence interval.

### Prognostic model analysis

**Figure 3:**
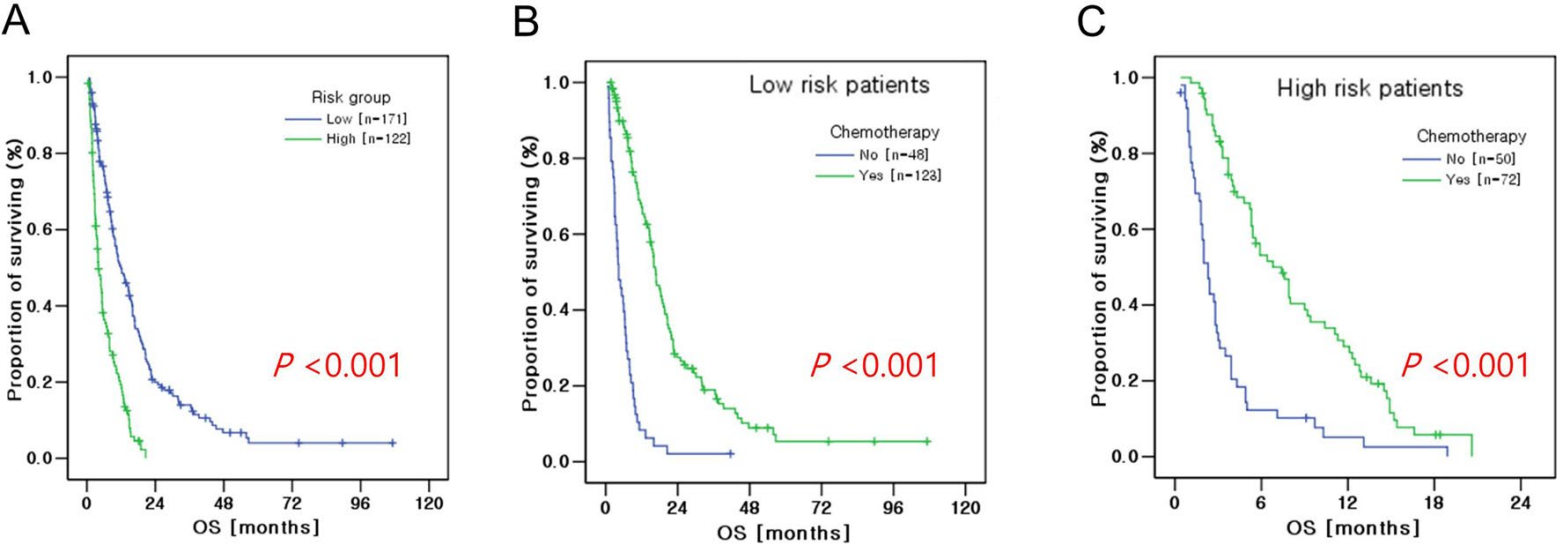
Kaplan–Meier overall survival (OS) curves according to risk group defined by number of adverse prognostic factors: (**A**) low-risk (0 to 2 adverse factors) and high-risk (3 to 4 adverse factors) groups, (**B**) palliative chemotherapy in the low-risk patients, and (**C**) palliative chemotherapy in the high-risk patients.

We divided patients into two subgroups based on four independent prognostic factors for OS: 171 patients with 0 to 2 adverse prognostic factors (low-risk group) and 122 patients with three to four adverse prognostic factors (high-risk group). These risk groups were significantly associated with OS (median OS for low- and high-risk groups: 11.9 months and 4.1 months, respectively, P < 0.001) (Fig. 3A). Regardless of the risk group, palliative chemotherapy had a benefit for OS in patients with bone metastasis. Among 171 low-risk patients, 123 (71.9%) patients received palliative chemotherapy. As shown in Fig. 3B, patients treated with palliative chemotherapy had longer OS than patients who received the best supportive care, including radiotherapy (median OS: 16.7 months vs. 4.3 months respectively, P < 0.001). Among 122 high-risk patients, OS was also longer in the palliative chemotherapy group (median OS: 6.8 months vs. 2.3 months, P < 0.001) (Fig. 3C).

## Discussion

Bone metastasis of CRC is rare, but it is related to SREs. Most patients have other organ metastasis and survival is 8.0 months in unselected patients. KRAS mutation affected different clinical manifestations in these patients. We showed that patients with KRAS mutation had a tendency to have shorter time to bone metastasis than patients with wild-type KRAS, but there were no significant survival differences between patients with KRAS wild-type and those with KRAS mutation. Several prognostic factors were noted in this study. Patients who received palliative chemotherapy showed prolonged survival compared to patients with supportive care, especially in patients with less adverse factors.

The observed frequency of bone metastasis in CRC was 1.2–12%, with a wide range^3,4^. Each study included CRC with bone metastasis in various clinical settings dealing with resected patients or metastatic patients. It might be related to the wide range of bone metastasis incidence in CRC patients. Recently, systemic chemotherapy was more effective and survival was prolonged. In addition, diagnostic modality such as positron emission tomography (PET) coupled with computed tomography (CT) scan (PET-CT) was developed and possibility of bone metastasis detection would be increased. Asymptomatic bone metastasis could be detected on routine follow-up. It might help prevent SREs, but it has a possibility of overtreatment, such as chemotherapy regimen change or radiotherapy. Therefore, the role of asymptomatic bone metastasis detection should be investigated in further studies. In contrast, surveillance cannot be performed until death in various circumstances, and routine follow-up schemes without bone scintigraphy or PET-CT may not detect bone metastasis. These factors are related to the underdiagnosis of bone metastasis.

In clinical practice, at the time of bone metastasis, multi-organ metastases were frequently observed. Bone-only metastasis was found in only 8.7% of subjects, and 91.3% of patients had other organ metastasis in this study. Commonly observed multi-organ metastases might be related to the poor prognosis in bone metastasis^5^. KRAS mutation has been studied as a metastasis promoting factor in CRC. The results of our study also showed that patients with KRAS mutation had a higher frequency of both lung and liver metastases at the time of bone metastasis. In addition, patients with KRAS mutation more frequently had high ALP and high CEA levels. Regarding KRAS mutation type (codon 12 vs. codon 13), there were no significant clinical differences between these two groups, except the NLR level.

The median time to bone metastasis was 27.2 months in metachronous patients. Time to bone metastasis was shorter in patients with KRAS mutation compared to patients with wild type KRAS, although it was not statistically significant. It has been reported that KRAS mutation can lead to shorter time to metastasis in liver, lung, and bone^17^. Initial CRC stage was also a significant factor for time to bone metastasis. Based on our data, first surveillance for identification of bone metastasis might be considered within 2 years after primary CRC diagnosis and attention is needed, especially in patients with initial stage 4. Additional evaluation and close monitoring 2 years after CRC diagnosis should also be performed.

Risk factors for bone metastasis in CRC have been reported in many studies^8^. Primary tumor location, tumor stage and histologic type were revealed as significant risk factors for bone metastasis. Regarding tumor location, other studies have shown that rectal cancer had a greater chance of bone metastasis than colon cancer. Although our studies showed more of a tendency for bone metastasis in rectal cancer compared to that in colon cancer, there was no significant difference between the two groups (P = 0.28). This study included all CRC patients treated in our institution, and the distribution of clinical factors, such as stage and histology, may have affected the results in the two groups.

The prognosis of bone metastasis in CRC patients was poor. The median survival of these patients was approximately 8 months^7,18^. Prognostic factors for bone metastasis in CRC have been evaluated in several studies. Multiple bone metastases, primary site of colon/lung metastasis, and elevated CEA level have been identified as poor prognostic factors. In our study, four prognostic factors were revealed: multi-organ metastasis, peritoneal metastasis, high NLR (≥ 2.7), and high ALP (≥ 123 IU/L). Of these factors, whether a patient had bone-only metastasis was a prognostic factor with the highest hazard ratio for OS (HR 2.43; 95% CI 1.39–4.25). The median survival was 7.8 months for patients with other organ metastasis and 20.4 months for those with bone-only metastasis. We also made a prognosis scoring model using the four independent factors. Risk group was categorized as low-risk (score 0–2) and high-risk (score 3–4) groups. The median survival of the low-risk group was 11.9 months. It was almost 1.5-fold (4 months longer) than that of unselected CRC patients with bone metastasis.

Bone metastasis is an important factor for quality of life and survival. Proper treatment, including radiotherapy, surgery and medical therapy, improves bone metastasis symptoms, such as pain, and bisphosphonates could delay the development of SREs^8^. In addition, bone metastasis has been known to be a poor prognostic factor in metastatic CRC. In our study, palliative chemotherapy provided patients a chance to increase survival up to 16.7 months in the low-risk group. The high-risk group also had a survival gain from palliative chemotherapy, although the survival benefit was less than the low-risk group. We had to pay attention to the interpretation of this survival data analyzed by the presence of chemotherapy, because patients who did not receive chemotherapy might have poor performance status or comorbidities, which might be related to poor prognosis. Our results suggest the possibility of chemotherapy benefit in bone metastasis patients, and predictive markers for chemotherapy should be further studied. A prognosis scoring model might help predict the survival for CRC patients with bone metastasis and guide treatment decisions, including palliative chemotherapy, or the best supportive care according to their benefits.

This study had several limitations. First, it was a retrospective study. Thus, the performance status could not be identified. Second, this study did not evaluate the impact of bisphosphonates on SREs. Bisphosphonates are not reimbursed in Korea. They are only permitted in hypercalcemia secondary to CRC. However, this study also has its strengths. Large numbers of patients were enrolled, and KRAS mutation status and tumor sidedness were analyzed to identify their roles in bone metastasis of CRC. In conclusion, bone metastasis of CRC is rare, but it is related to SREs. Most patients have other organ metastasis and survival is 8.0 months in unselected patients. It should receive more attention from clinicians. Time to bone metastasis and prognosis were not different according to KRAS mutation status in our study. Surveillance for bone metastasis might be considered in the first 2 years after CRC diagnosis, and additional evaluation and close monitoring 2 years after the CRC diagnosis should be performed. According to our prognosis scoring model, a patient’s prognosis could be predicted, and it may help to guide palliative chemotherapy in CRC with bone metastasis.

## Methods

### Patient selection

From January 2006 to April 2016, a total of 11,551 patients were diagnosed with CRC at Severance Hospital, Seoul, Korea. Diagnosis was made from surgical excision or tissue biopsy. Inclusion criteria were as follows: (1) age > 18 years, (2) histologically confirmed diagnosis of CRC, (3) radiologically or pathologically confirmed bone metastasis, and (4) available electronic medical records (including treatment information). Patients with other histology types, such as neuroendocrine tumors, small cell carcinoma, melanoma, or lymphoma, were excluded. A total of 321 patients fulfilled the inclusion and exclusion criteria. The Severance Hospital Institutional Review Board (IRB) approved this retrospective study, and the requirement to obtain informed consent was waived. This study was conducted in accordance with the Declaration of Helsinki and was consistent with Good Clinical Practice.

### Data collection

To diagnose bone metastasis, plain radiography, technetium-99m methylene diphosphonate (Tc-99m MDP) whole body bone scintigraphy, CT scan, PET-CT and magnetic resonance imaging (MRI) were performed. All available correlative radiographic studies were reviewed. The following baseline data were recorded at the time of bone metastasis: age, sex, tumor location, histologic type, initial stage, metastatic organ, and KRAS mutation status. For tumor sidedness determination, the splenic flexure was used for differentiation of the left side of the colon and right side of the colon. KRAS mutation status was identified by Sanger sequencing, the peptide nucleic acid (PNA)-mediated PCR clamping method^19^, or pyrosequencing. Sanger sequencing and the PNA-mediated PCR clamping method were used to detect mutations in codons 12 and 13. Pyrosequencing was performed to detect mutations at codons 12, 13, and 61. Metachronous bone metastasis was defined as the time to bone metastasis being greater than 3 months from initial CRC diagnosis.

Hematological and blood chemistry values included NLR, platelet count, ALP, and CEA. The NLR was calculated by dividing the absolute neutrophil count by the absolute lymphocyte count. Parameters related to bone metastasis included time to bone metastasis, site of bone metastasis, and SREs defined by pathologic fracture, spinal cord compression, surgery to bone, radiotherapy to bone, and hypercalcemia. After the diagnosis of bone metastasis, whether patients received palliative chemotherapy was also reviewed.

### Statistical analysis

The demographic and clinical characteristics were compared using the Chi square or Fisher exact test. The Mann–Whitney U test was used for comparisons of continuous variables. In patients with metachronous bone metastasis, freedom from bone metastasis since CRC diagnosis was estimated using the Kaplan–Meier method. Overall survival (OS) was defined as the time from the date of bone metastasis diagnosis to death from any cause. Hematological and blood chemistry values were initially recorded as continuous variables and later transformed into categorical variables according to lower or upper normal values (platelet count, ALP level, and CEA level) or the best cut-off point (NLR) determined by the Contal and O’Quigley method, which calculates the maximization of the hazard ratio based on log rank statistics and estimates the best cut-off value^20^. Survival curves were generated using the Kaplan–Meier method and compared using the log-rank test.

Univariate analysis was performed to determine associations of OS with the following prognostic factors: age; sex; tumor location and sidedness; timing of bone metastasis (synchronous or metachronous); histologic type; bone metastasis pattern (bone-only or not); liver, lung, peritoneal, and/or brain metastasis; NLR; ALP level; CEA level; KRAS mutation status; and palliative chemotherapy treatment. A stepwise multivariate analysis with Cox proportional hazard model was performed using significant factors from the univariate analysis. Hazard ratio (HR), 95% CI, and χ^2^ scores were obtained for all regressions. Statistical analyses were performed using PASW Statistics 18.0 (SPSS Inc., Chicago, IL, USA), SAS version 9.2 (SAS institute, Cary, NC, USA), and R version 3.1.3 (Institute for Statistics and Mathematics, Vienna, Austria www.R-project.org).

## Supplementary Information

Supplementary Information.

## Data Availability

Upon reasonable request, data and material are available from the corresponding authors.
