# Supplementary material for: Clinical features and KRAS mutation in colorectal cancer with bone metastasis
Source: Sci Rep. 2020 Dec 3;10:21180. doi: 10.1038/s41598-020-78253-x (PMC7713114; doi:10.1038/s41598-020-78253-x)
Supplement: Supplementary file 1 — Supplementary Information. [file 41598_2020_78253_MOESM1_ESM.docx]

**Supplementary information**

**Clinical features and KRAS mutation in colorectal cancer with bone metastasis**

Hyung Soon Park^1,2^, You Jin Chun^3^, Han Sang Kim^3^, Jee Hung Kim^4^, Choong-kun Lee^3^, Seung-Hoon Beom^3^, Sang Joon Shin^3^, Joong Bae Ahn^3^,*

^1^Department of Internal Medicine, College of Medicine, The Catholic University of Korea, Seoul, Korea; ^2^Division of Medical Oncology, Department of Internal Medicine, St. Vincent’s Hospital, The Catholic University of Korea, Suwon, Korea; ^3^Division of Medical Oncology, Department of Internal Medicine, Yonsei University College of Medicine, Seoul, Korea; ^4^Division of Medical Oncology, Department of Internal Medicine, Gangnam Severance Hospital, Yonsei University College of Medicine, Seoul, Korea

* Corresponding author:

Joong Bae Ahn, MD, PhD

Division of Medical Oncology, Department of Internal Medicine, Yonsei University College of Medicine, 50-1 Yonsei-Ro, Seodaemun-gu, Seoul, Korea, 03722, Phone: +82-2-2228-8130, Fax: +82-2-393-3652, E-mail: vvswm513@yuhs.ac

Supplementary Table 1. Clinical differences by KRAS mutation type

|  |  | KRAS | | | | |
| --- | --- | --- | --- | --- | --- | --- |
|  |  | Codon 12 mutation | | Codon 13 mutation | |  |
|  |  | No. (58) | % | No. (16) | % | P-value |
| Age | (median, range) | 62 | 28-77 | 64 | 42-79 | 0.895 |
| Sex | Male | 30 | 51.7 | 5 | 31.3 | 0.146 |
|  | Female | 28 | 48.3 | 11 | 68.8 |  |
| Primary site | Colon | 37 | 63.8 | 10 | 62.5 | 0.924 |
|  | Rectum | 21 | 36..2 | 6 | 37.5 |  |
| Tumor sideness | Left side | 40 | 70.2 | 12 | 75 | 1.000 |
|  | Right side | 17 | 29.8 | 4 | 25 |  |
| Metastasis pattern | Synchronous | 26 | 44.8 | 5 | 31.3 | 0.330 |
|  | Metachronous | 32 | 55.2 | 11 | 68.8 |  |
| Histologic type | Adenocarcinoma | 55 | 94.8 | 15 | 93.8 | 0.631 |
|  | SRC | 1 | 1.7 | 0 | 0 |  |
|  | Mucinous  carcinoma | 2 | 3.4 | 1 | 6.3 |  |
| Clinical stage at CRC diagnosis | 1 or 2 | 4 | 6.9 | 2 | 12.5 | 0.286 |
|  | 3 | 8 | 13.8 | 4 | 25 |  |
|  | 4 | 46 | 79.3 | 10 | 62.5 |  |
| Bone only metastasis | Yes | 1 | 1.7 | 0 | 0 | 1.000 |
|  | No | 57 | 98.3 | 16 | 100 |  |
| Other organ metastasis |  |  |  |  |  |  |
| Liver metastasis | No | 16 | 27.6 | 4 | 25 | 1.000 |
|  | Yes | 42 | 72.4 | 12 | 75 |  |
| Lung metastasis | No | 22 | 37.9 | 3 | 18.8 | 0.151 |
|  | Yes | 36 | 62.1 | 13 | 81.3 |  |
| Peritoneal metastasis | No | 44 | 75.9 | 13 | 81.3 | 0.750 |
|  | Yes | 14 | 24.1 | 3 | 18.8 |  |
| Brain metastasis | No | 53 | 91.4 | 14 | 87.5 | 0.640 |
|  | Yes | 5 | 8.6 | 2 | 12.5 |  |
| Site of bone metastasis |  |  |  |  |  |  |
| Spine | No | 21 | 36.2 | 5 | 31.3 | 0.713 |
|  | Yes | 37 | 63.8 | 11 | 68.8 |  |
| Pelvis | No | 28 | 48.3 | 9 | 56.3 | 0.572 |
|  | Yes | 30 | 51.7 | 7 | 43.8 |  |
| Long bone | No | 46 | 79.3 | 13 | 81.3 | 1.000 |
|  | Yes | 12 | 20.7 | 3 | 18.8 |  |
| Other bone^a^ | No | 36 | 62.1 | 11 | 68.8 | 0.623 |
|  | Yes | 22 | 37.9 | 5 | 31.3 |  |
| Laboratory values  at diagnosis of bone metastasis  (median, range) | NLR | 4.1 | 0.8-31.8 | 2 | 0.9-15.3 | 0.034 |
|  | Platelet (x1,000/uL) | 253 | 25-654 | 243 | 87-443 | 0.926 |
|  | ALP (IU/L) | 136 | 42-1,509 | 101 | 32-374 | 0.171 |
|  | CEA (ng/mL) | 166.6 | 2.3-20,000 | 142.8 | 3.2-2,039.8 | 0.350 |
| Skeletal related event | No | 19 | 32.8 | 3 | 18.8 | 0.364 |
|  | Yes | 39 | 67.2 | 13 | 81.3 |  |

Abbreviations: CRC, colorectal cancer; CTx, chemotherapy; NLR, neutrophil-lymphocyte ratio; SRC, signet ring cell carcinoma; ALP, alkaline phosphatase; CEA, carcinoembryonic antigen; No, number

^a^Other bone include skull, rib, scapula, knee, clavicle, sternum

Supplementary Table 2. Clinical differences by primary tumor sideness

|  |  | Primary tumor sideness | | | | |
| --- | --- | --- | --- | --- | --- | --- |
|  |  | Left side | | Right side | |  |
|  |  | No | % | No. | % | P-value |
| Age | (median, range) | 60 | 28-87 | 65 | 32-79 | 0.036 |
| Sex | Male | 158 | 63.2 | 25 | 39.7 | 0.001 |
|  | Female | 92 | 36.8 | 38 | 60.3 |  |
| KRAS mutation | Wild type | 105 | 65.6 | 19 | 46.3 | 0.023 |
|  | Mutant type | 55 | 34.4 | 22 | 53.7 |  |
| Metastasis pattern | Synchronous | 107 | 42.8 | 34 | 54.0 | 0.111 |
|  | Metachronous | 143 | 57.2 | 29 | 46.0 |  |
| Histologic type | Adenocarcinoma | 229 | 91.6 | 55 | 87.3 | 0.530 |
|  | SRC | 10 | 4 | 4 | 6.3 |  |
|  | Mucinous carcinoma | 11 | 4.4 | 4 | 6.3 |  |
| Clinical stage at CRC diagnosis | 1 or 2 | 17 | 6.9 | 2 | 3.2 | 0.145 |
|  | 3 | 58 | 23.6 | 9 | 14.5 |  |
|  | 4 | 171 | 69.5 | 51 | 82.3 |  |
| Bone-only metastasis | Yes | 25 | 10 | 3 | 4.8 | 0.193 |
|  | No | 225 | 90 | 60 | 95.2 |  |
| Other organ metastasis | |  |  |  |  |  |
| Liver metastasis | No | 116 | 46.4 | 19 | 30.2 | 0.02 |
|  | Yes | 134 | 53.6 | 44 | 69.8 |  |
| Lung metastasis | No | 113 | 45.2 | 37 | 58.7 | 0.055 |
|  | Yes | 137 | 54.8 | 26 | 41.3 |  |
| Peritoneal metastasis | No | 198 | 79.2 | 38 | 60.3 | 0.002 |
|  | Yes | 52 | 20.8 | 25 | 39.7 |  |
| Brain metastasis | No | 239 | 95.6 | 59 | 93.7 | 0.513 |
|  | Yes | 11 | 4.4 | 4 | 6.3 |  |
| Site of bone metastasis | |  |  |  |  |  |
| Spine | No | 79 | 31.6 | 17 | 27 | 0.478 |
|  | Yes | 171 | 68.4 | 46 | 73 |  |
| Pelvis | No | 122 | 48.8 | 29 | 46 | 0.694 |
|  | Yes | 128 | 51.2 | 34 | 54 |  |
| Long bone | No | 200 | 80 | 47 | 74.6 | 0.348 |
|  | Yes | 50 | 20 | 16 | 25.4 |  |
| Other bone^a^ | No | 156 | 62.4 | 35 | 55.6 | 0.319 |
|  | Yes | 94 | 37.6 | 28 | 44.4 |  |
| Laboratory values  at diagnosis of bone metastasis  (median, range) | NLR | 3.5 | 0.4-31.8 | 3.5 | 0.9-13.7 | 0.993 |
|  | Platelet (x1,000/uL) | 247 | 25-637 | 254 | 67-654 | 0.963 |
|  | ALP (IU/L) | 109 | 17-1,338 | 147 | 42-1,509 | 0.007 |
|  | CEA (ng/mL) | 70.4 | 0.4-20,000 | 109.6 | 1.4-18,053 | 0.110 |
| Skeletal related event | No | 89 | 35.6 | 29 | 46 | 0.127 |
|  | Yes | 161 | 64.4 | 34 | 54 |  |

Abbreviations: CRC, colorectal cancer; NLR, neutrophil-lymphocyte ratio; SRC, signet ring cell carcinoma; ALP, alkaline phosphatase; CEA, carcinoembryonic antigen; No, number

^a^Other bone include skull, rib, scapula, knee, clavicle, sternum
